# Organizational culture and turnover intention among Generation Z in Korea: Associations with job satisfaction and organizational commitment

**DOI:** 10.3389/fpsyg.2026.1820390

**Published:** 2026-06-26

**Authors:** Yeji Nam, Nayoung Kim, Giyun Kim, Sehee Hong

**Affiliations:** Department of Education, Korea University, Seoul, Republic of Korea

**Keywords:** Generation Z, job satisfaction, Korean labor market, organizational commitment, organizational culture, person-organization fit theory, turnover intention

## Abstract

This study examines the relationship between organizational culture and turnover intention among Korean Generation Z employees, addressing the widely held perception that they prefer organizational cultures different from those of previous generations. Drawing on Person–Organization (P–O) Fit theory, the study explores how different types of organizational culture are associated with turnover intention directly and indirectly through job satisfaction and organizational commitment. By focusing on these relationships within a Generation Z sample, this study aims to provide exploratory insights into how organizational culture is related to employee attitudes and turnover intention in the Korean context. To test these relationships, data from the third year (2022) of the second wave of the Human Capital Corporate Panel II (HCCP II), developed by the Korea Research Institute for Vocational Education and Training (KRIVET), were analyzed. Specifically, 456 individuals born between 1995 and 2004 (Generation Z) were included in the analysis. The results indicate that an adhocracy culture was positively associated with job satisfaction and organizational commitment, while showing a negative association with turnover intention. A clan culture was also positively associated with job satisfaction and organizational commitment, while a hierarchy culture was positively associated with job satisfaction. In contrast, a market culture showed a limited association, being positively related only to organizational commitment. In addition, the indirect paths to turnover intention via job satisfaction and organizational commitment were not statistically significant. These findings provide exploratory insights into the associations among organizational culture, job satisfaction, organizational commitment, and turnover intention among Korean Generation Z employees.

## Introduction

As generational shifts continue to reshape organizational landscapes, Generation Z—defined as individuals born after 1995—has become a critical focus in modern recruitment and talent management strategies (Bazatova and Nilsson, 2024). Given that this cohort represents a growing segment of the global workforce, organizations are increasingly exploring human resource strategies that align with their distinct workplace values, such as an emphasis on continuous career growth and psychological safety. Accordingly, understanding how these employees perceive their organizational environments has become a relevant area of inquiry.

In Korea, high turnover intention observed among younger employees has attracted growing attention. According to the Korea Employers Federation (2024), 83.2% of employees in their twenties reported considering a job change, exceeding the overall average of 69.5%. Such passive withdrawal often precedes more overt forms of disengagement, such as tardiness, absenteeism, and counterproductive work behaviors (Kim and Kao, 2014), all of which undermine organizational performance and culture. While various factors may contribute to this phenomenon, there is a particular need to examine the environmental factors within the organization that may be associated with these trends. Recent empirical evidence in the South Korean context further demonstrates that the turnover motivations of the younger workforce are not merely rooted in individual dispositions, but are complexly shaped by structural conditions and organizational environments (Lee and Suh, 2025).

Organizational culture is potentially associated with various employee outcomes, including job satisfaction (Carvalho et al., 2018) and organizational commitment (Bhatti et al., 2016). In turn, these factors are recognized as variables that may relate to turnover intention (Alam and Asim, 2019). This suggests that different cultural perceptions might share exploratory links with turnover intention, possibly through indirect associations involving satisfaction and commitment. From the perspective of person–organization fit (P–O fit), these relationships may be explained by the degree of compatibility between employees and their organizational environments. P–O fit suggests that such compatibility is associated with favorable work attitudes and lower turnover intention (Kristof, 1996). Therefore, the P–O fit perspective provides a useful theoretical lens for understanding how organizational culture may relate to employee attitudes among Generation Z employees.

Despite these discussions, empirical evidence regarding the perceived influence of organizational culture among Korean Generation Z employees remains limited. Specifically, more research is needed to explore how specific cultural archetypes operate within the unique psychological expectations of the Korean youth workforce (Jang and Lee, 2024). While this study does not compare Generation Z with other cohorts, it seeks to provide a focused, exploratory analysis of the relationships observed within this specific group.

Accordingly, the present study seeks to exploratively examine the potential associations between organizational culture and turnover intention among Generation Z in Korea. By analyzing the indirect links of job satisfaction and organizational commitment, this study aims to provide preliminary and association-based insights into how the perceived values of the sampled workforce interact with organizational environments. The findings are intended to offer foundational insights for developing more nuanced human resource strategies.

## Theoretical review

### Emergence of Generation Z and their turnover intentions

While definitions may vary, Generation Z is generally defined as individuals born after 1995 (Barrio, 2022). Globally, this generation anticipated to represent a significant segment of future labor markets. In Korea, Generation Z comprises approximately 16.6% of the population and is rapidly emerging as a key workforce segment (Korea Insurance Research Institute, 2023). This has led to increased efforts within the Korean labor market to explore the professional characteristics of this cohort.

Having grown up in a digital environment, Korean Generation Z is often characterized as being highly proficient with technology and social media (Anh Do et al., 2023), and is recognized as one of the most highly educated cohorts (Fry and Parker, 2018). Literature suggests that they may prefer work environments that promote growth, autonomy, and diverse experiences (Rue, 2018).

These perceived attributes may relate to a preference for diverse roles and career mobility, rather than committing long-term to a single organization. This tendency is reflected in the high turnover intentions frequently observed within this cohort in the Korean labor market, potentially driven by a desire for organizational cultures that are perceived to better align with their values (Jang and Lee, 2024). Accordingly, Korean companies have begun adopting tailored communication strategies and mentoring programs to better engage and retain Generation Z employees (Hankyung, 2024). However, to further address turnover, it may be useful to look beyond surface-level characteristics and examine the alignment between the perceived values of this cohort and their organizational environments. Grounded in quantitative data of the Korean youth workforce, recent literature emphasizes that subjective perceptions regarding career growth potential, work-life balance, and welfare systems serve as critical determinants that significantly heighten their turnover deliberations when expectations are unmet (Lee and Suh, 2025).

### Organizational culture

The high turnover intention among Generation Z may be discussed in light of reported shifts in value orientations. Some literature suggests a transition from traditional collectivism toward a greater emphasis on individual authenticity and personal satisfaction (Törocsik et al., 2014). Some studies have suggested that younger employees may place relatively greater emphasis on workplace experiences and well-being (McCrindle and Wolfinger, 2009). These perspectives have also been discussed in relation to preferences for psychological safety and value alignment in organizational settings (Twenge, 2023). Consequently, a perceived misalignment between these potential expectations and traditional, rigid organizational structures may relate to turnover intentions. This underscores the relevance of examining how different types of organizational culture associate with the job satisfaction and commitment of the sampled Generation Z employees.

Organizational culture is a multidimensional construct encompassing the shared values, beliefs, norms, and behavioral patterns among members of an organization. It has been associated with organizational identity, stability, and performance outcomes, ensuring stability, and influencing performance outcomes (Tsai, 2011). Depending on the organization's goals and characteristics, organizational culture can be classified into various types. One of the most prominent frameworks, the Competing Values Model (CVM), categorizes organizational culture along two axes: flexibility and discretion (positive y-axis) vs. stability and control (negative y-axis), and external focus and differentiation (positive x-axis) vs. internal focus and integration (negative x-axis), resulting in four distinct cultural types (Cameron and Quinn, 2011).

The first quadrant represents adhocracy culture, which emphasizes creativity, innovation, and agility in responding to uncertain environments. The second quadrant, clan culture, focuses on collaboration and participation, fostering trust and cohesion among members and enhancing loyalty and morale. The third quadrant, hierarchy culture, values stability and predictability, relying on formal procedures and rules to achieve organizational efficiency. Lastly, the fourth quadrant, market culture, centers on performance and goal attainment, encouraging internal competition and emphasizing fair evaluation and compensation based on individual achievement (Cameron et al., 2006). For Generation Z, the perceived distinction between these types may be relevant; while they might perceive hierarchical control as restrictive, they may potentially respond more favorably to the merit-based, result-oriented nature often associated with market culture. This aligns with recent empirical evidence confirming that a perceived lack of alignment with their expectations within the organizational environment serves as a critical determinant of the younger workforce's long-term career decisions (Lee and Suh, 2025).

To provide a rigorous theoretical foundation for these interconnected variables, this study draws upon the person–organization fit (P–O fit) perspective as a theoretical lens for understanding how organizational culture may relate to employee attitudes. P-O fit is defined as the compatibility between individuals and organizations, which occurs when their values, goals, and core characteristics are mutually aligned (Kristof, 1996). In this context, organizational culture functions as the definitive environmental landscape that establishes the shared values and behavioral expectations within the firm (Chatman, 1989).

According to P-O fit theory, when employees perceive a high degree of congruence between their personal values and the prevailing organizational culture, they experience enhanced psychological comfort, leading to positive work attitudes such as elevated job satisfaction and stronger organizational commitment (Cable and DeRue, 2002; Hoffman and Woehr, 2006). Conversely, a cultural mismatch generates cognitive dissonance, which acts as a powerful antecedent to turnover intention (Verquer et al., 2003). Therefore, the P–O fit perspective provides a useful framework for interpreting how different organizational culture types may be associated with employee attitudes and turnover intention among Generation Z employees.

### The relationships among organizational culture, job satisfaction, organizational commitment, and turnover intention

Guided by P-O Fit Theory (Kristof, 1996), which proposes that the congruence between an individual's values, goals, and personality and those of the organization shapes key work-related attitudes and behaviors, this section examines the associations among organizational culture, job satisfaction, organizational commitment, and turnover intention among Generation Z employees in Korea. When employees perceive a strong fit between their personal values and the prevailing organizational culture, they are more likely to experience higher job satisfaction and organizational commitment, and less likely to intend to leave the organization.

#### Organizational culture and turnover intention

Turnover intention is often regarded as a proximal predictor of actual turnover behavior. Previous studies have suggested that organizational culture may be associated with employees' turnover intention by shaping their perceptions of the work environment and their attachment to the organization (Cronley and Kim, 2017; Park and Kim, 2009). From a P–O fit perspective, employees who perceive a mismatch between their expectations and organizational culture may be more likely to consider leaving the organization, whereas those who perceive greater compatibility may be less likely to do so (Kristof, 1996). Although recent studies have begun examining turnover among Generation Z through the lens of organizational culture (e.g., Cho et al., 2024), empirical evidence remains limited. Accordingly, the following hypothesis is proposed:

*H1*. Different types of organizational culture are associated with turnover intention among Korean Generation Z employees.

#### Organizational culture and job satisfaction

Job satisfaction is defined as both a sense of accomplishment through work (McCormick and Tiffin, 1974) and a positive emotional state resulting from job experiences (Locke, 1976). Although findings vary across cultural and organizational contexts (Carvalho et al., 2018; Lund, 2003; Rawashdeh et al., 2015), organizational culture is consistently identified as a significant correlate of job satisfaction (Habib et al., 2014). For example, Lund (2003) found that clan and adhocracy cultures were linked to higher job satisfaction in U.S. marketing firms, whereas market and hierarchy cultures showed a negative association. In contrast, Rawashdeh et al. (2015) reported that only clan culture was positively related to job satisfaction in a Jordanian airline. Despite contextual differences, these studies support the notion that organizational culture is closely related to job satisfaction (Choi et al., 2008). More recently, Cho et al. (2024) demonstrated that flexible and collaborative cultural orientations were positively associated with satisfaction among younger Korean workers, suggesting that culture types aligned with Generation Z values may foster greater fit and satisfaction. Based on these findings, the following hypothesis is proposed:

*H2*. Different types of organizational culture are associated with job satisfaction among Korean Generation Z employees.

#### Organizational culture and organizational commitment

Organizational commitment, defined as emotional attachment to and identification with the organization (Meyer and Allen, 1991), is widely recognized as an important employee attitude. Previous studies have suggested that various organizational culture types, including clan, hierarchy, adhocracy, and market cultures, may be associated with higher levels of organizational commitment (Carvalho et al., 2018; Nikpour, 2017), although the evidence remains fragmented and often limited to specific industries and organizational contexts. More recently, Chen et al. (2026) found that person–organization value fit was positively associated with organizational commitment among Millennial and Generation Z employees. In addition, organizational characteristics that promote employees' sense of belonging and growth have been linked to stronger organizational commitment. These findings suggest that organizational culture may be associated with organizational commitment among Korean Generation Z employees. Accordingly, the following hypothesis is proposed:

*H3*. Different types of organizational culture are associated with organizational commitment among Korean Generation Z employees.

#### Job satisfaction and organizational commitment

Job satisfaction and organizational commitment are closely interrelated constructs. Employees who experience higher levels of job satisfaction are more likely to develop stronger emotional attachment to and identification with their organization, resulting in higher organizational commitment (Mosadeghrad et al., 2008). Previous studies have consistently reported a positive relationship between job satisfaction and organizational commitment across various organizational settings, suggesting that favorable work experiences contribute to stronger organizational attachment. Based on these findings, the following hypothesis is proposed:

*H4*. Job satisfaction is associated with organizational commitment among Korean Generation Z employees.

#### Job satisfaction, organizational commitment, and turnover intention

Job satisfaction and organizational commitment have been widely recognized as important antecedents of turnover intention. Previous studies have consistently reported that employees who are more satisfied with their jobs and more committed to their organizations are less likely to consider leaving their current workplace (Alam and Asim, 2019; Guzeller and Celiker, 2020). In the Korean context, Park (2024) further found that job satisfaction partially explained the relationships between several organizational culture types and turnover intention among public-sector employees. Although empirical evidence remains limited for Korean Generation Z employees, existing findings suggest that both job satisfaction and organizational commitment may be negatively associated with turnover intention. Accordingly, the following hypotheses are proposed:

*H5*. Job satisfaction is negatively associated with turnover intention among Korean Generation Z employees.

*H6*. Organizational commitment is negatively associated with turnover intention among Korean Generation Z employees.

In addition, this study exploratorily examines potential indirect associations among organizational culture, job satisfaction, organizational commitment, and turnover intention.

## Research methods

### Data

This study utilized data from the third year (2022) of the second wave of the Human Capital Corporate Panel II (HCCP II), developed by the Korea Research Institute for Vocational Education and Training (KRIVET). To investigate the relationship between organizational culture and turnover intention among Korean Generation Z, 456 employees born between 1995 and 2004 were selected for analysis.

The HCCP II dataset was appropriate for this study for two main reasons. First, it includes key variables related to organizational culture, job satisfaction, and organizational commitment, allowing for a comprehensive analysis of Generation Z employees‘ workplace perceptions. Second, its large-scale national design supports broader analyses of organizational experiences within the Korean labor market. Specifically, the second wave of HCCP II has annually tracked workers in companies with 100 or more employees since 2020. In 2022, the dataset comprised 9,512 employees: 5,674 from firms with 100–299 employees, 2,976 from firms with 300–999 employees, and 862 from firms with 1,000 or more employees. This large-scale and systematically collected dataset provided a solid foundation for analyzing Korean Generation Z employees' organizational experiences.

Table 1 presents the demographic and organizational characteristics of the sample. The respondents consisted of Korean Generation Z employees, with the majority born in the 1990s, reflecting the early segment of Generation Z represented in the dataset. Additionally, the mean age of participants was 25.44 years (SD = 2.01), and the average organizational tenure was 1.98 years (SD = 1.87). Age and tenure were calculated using birth year and company entry year information provided in the HCCP II dataset.

**Table 1:** Sample characteristics (*N* = 456).

Variable	Category	N (%)	Mean (SD)
Gender	Male	214 (46.93)	-
	Female	242 (53.07)	-
Birth year	1990s (1995–1999)	413 (90.57)	-
	2000s (2000–2004)	43 (9.43)	-
Education	General high school	51 (11.18)	-
	Vocational high school	160 (35.09)	-
	Junior college	68 (14.91)	-
	Bachelor's degree	176 (38.60)	-
	Master's degree	1 (0.22)	-
Employment status	Regular	412 (90.35)	-
	Non-regular	44 (9.65)	-
Industry	Manufacturing	334 (73.25)	-
	Financial	16 (3.51)	-
	Non-financial	106 (23.24)	-
Firm size	Small (100–299 employees)	301 (66.01)	-
	Medium (300–999 employees)	122 (26.75)	-
	Large (≥1000 employees)	33 (7.24)	-
Job position	Staff-level employees	388 (85.09)	-
	Assistant manager/Senior staff	51 (11.18)	-
	Manager-level or above	17 (3.73)	-
Age		-	25.44 (2.01)
Tenure (Years)		-	1.98 (1.87)

The sample includes only firms with 100 or more employees and selected industry categories available in the dataset. Age and tenure were calculated using 2022 as the reference year.

It should be noted that the sample characteristics reflect the design of the HCCP II dataset. The HCCP II collects data from firms with 100 or more employees. In addition, the survey covers selected industries based on the Korean Standard Industrial Classification (KSIC), including manufacturing (C), financial and insurance activities (K), information and communication (J), professional, scientific, and technical activities (M), education (P), and arts, sports, and recreation-related services (R). However, the publicly available dataset provides industry information in aggregated categories (manufacturing, financial, and non-financial sectors). Therefore, the findings should be interpreted within the scope and structure of the dataset.

### Measurement tools

The measurement items used in this study were obtained directly from the HCCP II survey developed by the KRIVET. As this study relied on secondary survey data, the measurement instruments were based on the publicly available HCCP II survey items. However, detailed information regarding the original development process of the measurement items was not publicly available in the HCCP II documentation.

#### Organizational culture

Organizational culture was measured using the Competing Values Framework established by Cameron and Quinn (2011). This framework classified organizational culture into four types: Adhocracy Culture, Clan Culture, Hierarchy Culture, and Market Culture. Each category was operationalized through a set of three items in the survey that captured the defining attributes of the respective cultural types. All items were measured using a five-point Likert scale, with response options ranging from ‘Strongly Disagree' (1) to ‘Strongly Agree' (5) and the details were presented in Table 2.

**Table 2:** Item description for the constructs.

Constructs	Item
Adhocracy culture	(1) Our company encourages change and new initiatives.
	(2) Our company provides appropriate rewards for innovation.
	(3) Our company values and rewards creative individuals more than diligent ones.
Clan culture	(4) Our company fosters a family-like organizational atmosphere.
	(5) Our company emphasizes harmony and a sense of unity.
	(6) Our company places great importance on teamwork.
Hierarchy culture	(7) Our company emphasizes formal procedures, rules, and policies.
	(8) Our company has a top-down communication and information flow.
	(9) Our company emphasizes a sense of hierarchy between supervisors and subordinates.
Market culture	(10) Our company emphasizes a competitive atmosphere and performance achievement.
	(11) Our company values the expertise and skills necessary for task execution.
	(12) Our company evaluates employees based on job performance and results.
Organizational commitment	(1) I perceive the company's issues as my own.
	(2) If I decide to leave this company, I would lose too much in my life.
	(3) This company is worth my loyalty.
Job satisfaction	(1) I am satisfied with the content of my current job.
	(2) I am satisfied with my current salary. (3) I am satisfied with interpersonal relationships in my workplace.
Turnover intention	(1) I would consider moving to another company if offered even slightly better conditions.

Measurement items were obtained from the Human Capital Corporate Panel II (HCCP II) survey developed by the Korea Research Institute for Vocational Education and Training (KRIVET).

#### Organizational commitment

Organizational commitment was measured by three items asking the survey respondents to assess how much they were committed to their companies. All items were rated on a five-point Likert scale, with response options ranging from 'Strongly Disagree' (1) to 'Strongly Agree' (5). Higher scores indicate higher levels of organizational commitment. In this study, organizational commitment was operationalized as an overall construct, capturing the general level of employees' attachment to the organization rather than distinguishing between specific sub-dimensions. The details of the three items were shown in Table 2.

#### Job satisfaction

Job satisfaction was assessed across three dimensions: job content, salary, and interpersonal relationships. Each dimension was measured using a single item on a five-point Likert scale. The response options ranged from ‘Strongly Disagree' (1) to ‘Strongly Agree' (5), so higher scores mean that the survey respondents had greater job satisfaction with their companies. The details of the three items were presented in Table 2.

#### Turnover intention

Turnover intention was measured by a single-item statement: “I would consider moving to another company if offered even slightly better conditions.” Responses were recorded on a five-point Likert scale, with higher scores reflecting an increased likelihood of turnover intention.

Although multi-item scales are generally recommended, turnover intention is widely conceptualized as a proximal and unidimensional construct that captures an individual's conscious and deliberate willingness to leave. Therefore, it can be appropriately assessed using a single, direct item without substantial loss of validity. In addition, given the prediction-oriented nature of PLS-SEM, the use of single-item constructs is considered acceptable when the concept is concrete and clearly defined (Hair et al., 2022). Accordingly, the use of a single-item measure in this study is both methodologically and theoretically justified. The item referring to perceived loss associated with leaving the organization was modeled as part of organizational commitment instead, specifically continuance commitment, as it reflects the perceived cost of leaving rather than the intention to leave itself (Meyer and Allen, 1991). This approach preserves conceptual clarity between constructs.

### Analytical method

This study explored associations among organizational culture, job satisfaction, organizational commitment, and turnover intention among Korean Generation Z employees. Organizational culture was treated as the independent variable, turnover intention as the dependent variable, while job satisfaction and organizational commitment were included as related variables within the structural model to explore potential indirect associations. To analyze these relationships, Partial Least Squares Structural Equation Modeling (PLS-SEM) was employed using SPSS Statistics 29.0 and SmartPLS 3.

PLS-SEM estimates partial regression relationships using Ordinary Least Squares (OLS) and Principal Components Analysis (PCA), allowing the maximization of explained variance while minimizing residual error (Hair et al., 2022). Unlike Covariance-Based SEM (CB-SEM), PLS-SEM does not require multivariate normality and flexibly accommodates complex model specifications, including reflective and formative constructs as well as higher-order constructs.

The choice of PLS-SEM aligned with the study's analytical needs. First, this study adopts a prediction-oriented approach aimed at examining relationships among constructs rather than testing a fully established theoretical model, which aligns with the strengths of PLS-SEM. Second, the model includes a formative construct, as job satisfaction was conceptualized as being formed by multiple distinct components (i.e., job content, salary, and interpersonal relationships). These components are not necessarily interchangeable and may not be highly correlated, but each represents a unique aspect contributing to the overall construct. This specification is well suited to the PLS-SEM framework. Third, PLS-SEM allows for greater flexibility in handling complex model structures and measurement specifications. In this study, constructs such as turnover intention were operationalized using direct measures, consistent with prior research and the conceptualization of the construct as concrete and unidimensional. The use of single-item constructs is available within the context of PLS-SEM, as it is primarily prediction-oriented and places fewer restrictions on measurement models compared to CB-SEM (Hair et al., 2022). Fourth, the study included a higher-order construct specification for hierarchy culture. Specifically, hierarchy culture was modeled as a higher-order construct composed of two sub-dimensions: norm-oriented and rank-oriented culture. Further details are reported in the Results section.

For analyzing the higher-order construct, the disjoint two-stage approach was applied. In the first stage, lower-order constructs were estimated; in the second stage, these results were used to estimate the higher-order construct. This method has been shown to provide stable and accurate estimates (Becker et al., 2023).

The PLS-SEM analysis proceeded in two stages: measurement model evaluation and structural model evaluation. For reflective constructs, outer loadings greater than 0.708 indicated acceptable reliability, while Cronbach's alpha and composite reliability between 0.60 and 0.90 suggested internal consistency. Convergent validity was confirmed through an AVE (Average Variance Extracted) ≥0.50. Discriminant validity was assessed using the Fornell-Larcker criterion and HTMT, with thresholds of AVE's square root > inter-construct correlations and HTMT < 0.85, respectively (Hair et al., 2022).

For formative constructs, three criteria were used: convergent validity, multicollinearity, and significance of outer weights. Convergent validity was tested through the correlation of job satisfaction indicators with an overall satisfaction item from the same questionnaire. Multicollinearity was assessed using Variance Inflation Factor (VIF) values, with VIF < 3 indicating no serious issues. The significance of outer weights for each indicator was also tested via bootstrapping.

The structural model was evaluated using effect size (*f*^2^), coefficient of determination (*R*^2^), predictive relevance (*Q*^2^), and Root Mean Square Error (RMSE). Effect sizes of 0.35, 0.15, and 0.02 were interpreted as large, medium, and small, respectively (Cohen, 1988). *Q*^2^ > 0 indicated sufficient predictive relevance. RMSE values of the PLS-SEM model were compared with those of a general linear regression model; a lower RMSE in PLS-SEM signified superior predictive accuracy. The analysis also examined direct, indirect, and total effects, with bias-corrected 95% bootstrapping confidence intervals used to test significance.

**Figure 1:**
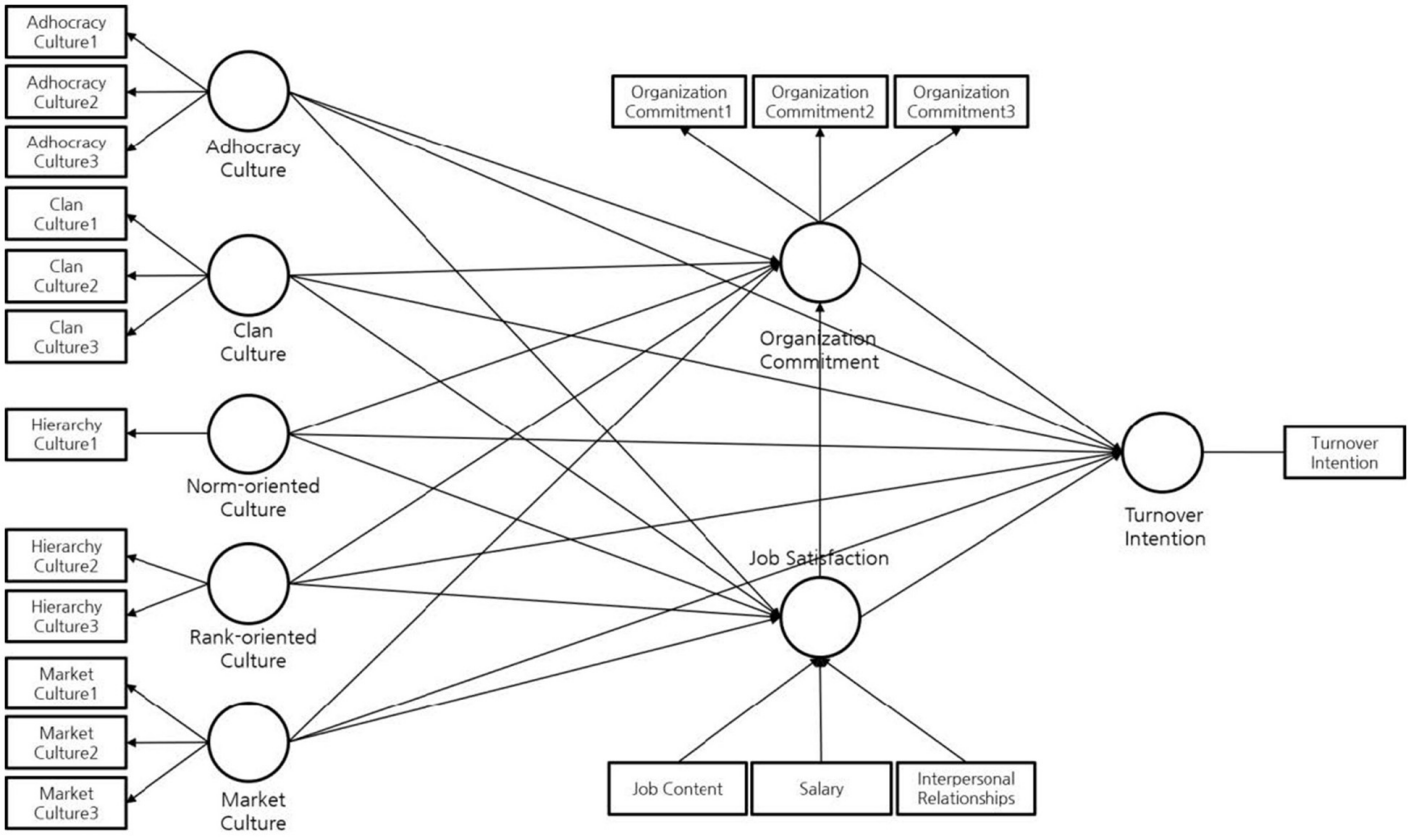
Research model (first-order).

**Figure 2:**
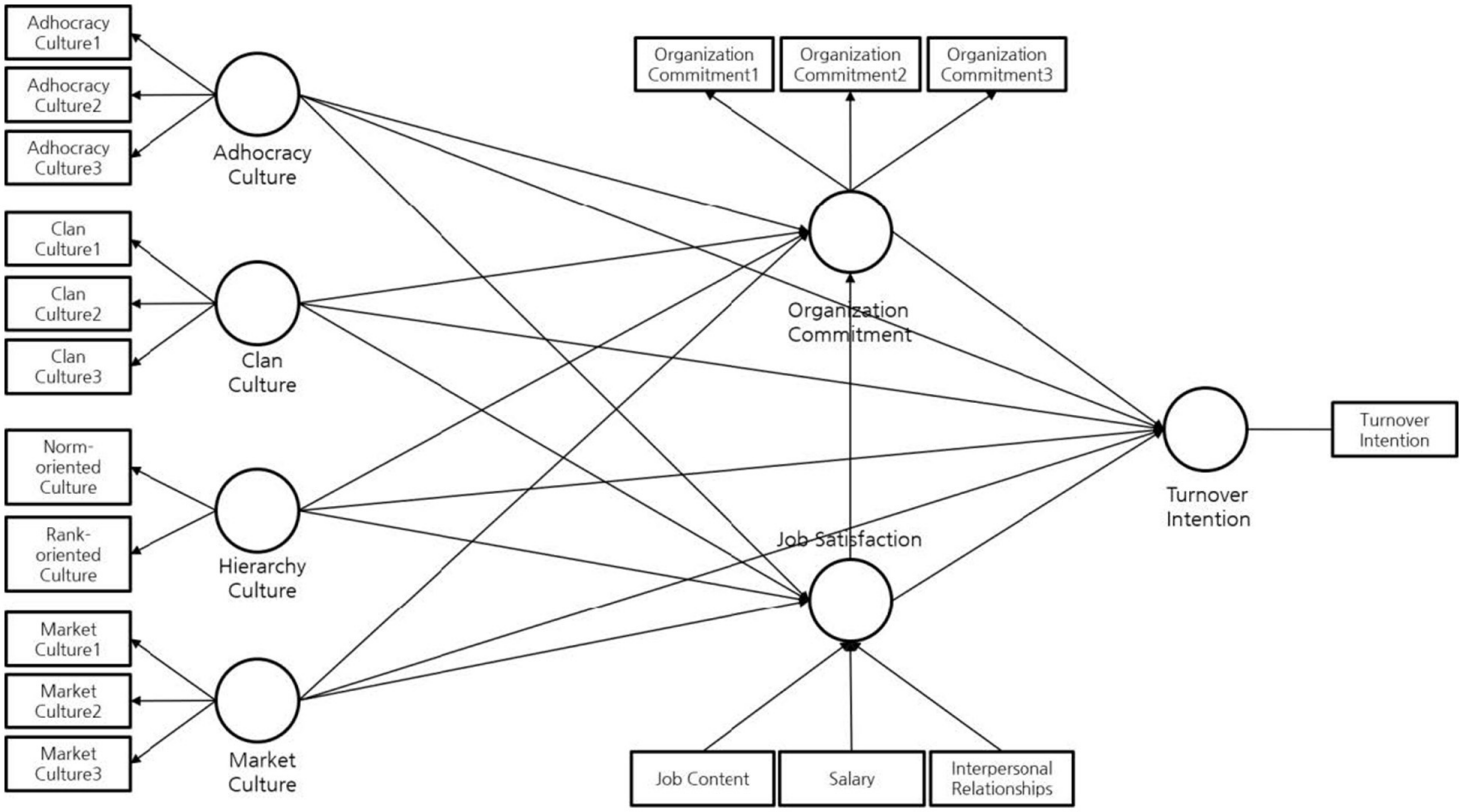
Research model (second-order).

Based on the analytical methods described above, the research model developed in this study is illustrated in Figure 1 and Figure 2, where Figure 1 represents the first-order model, and Figure 2 represents the second-order model. Before proceeding with the analysis, Harman's single-factor test was examined to assess potential common method variance. The results indicated that the first factor accounted for 31.76% of the total variance, which is below the recommended threshold of 50%, suggesting that common method variance is unlikely to be a serious concern (Kock, 2021). Finally, although the data have a nested structure in which employees are clustered within firms, the average number of respondents per firm was relatively small (M = 1.99). Moreover, most firms included only one or two respondents, limiting the ability to reliably estimate between-firm variance and cluster-level effects. Therefore, multilevel analysis was not considered appropriate in the present study.

## Results

### Descriptive statistics and correlation analysis

Descriptive statistics and correlation analysis for key constructs are provided in Table 3.

**Table 3:** Descriptive statistics and correlation analysis for key constructs.

Constructs	1	2	3	4	5	6	Mean	SD	Kurtosis	Skewness
1. Adhocracy Culture							0.000	1.000	0.177	−0.205
2. Clan Culture	0.624^***^						0.000	1.000	0.433	−0.250
3. Hierarchy Culture	0.411^***^	0.493^***^					0.000	1.000	0.368	−0.499
4. Market Culture	0.536^***^	0.523^***^	0.481^***^				0.000	1.000	0.605	−0.090
5. Job Satisfaction	0.498^***^	0.536^***^	0.435^***^	0.441^***^			0.000	1.000	−0.059	0.050
6. Organization Commitment	0.481^***^	0.484^***^	0.222^***^	0.379^***^	0.511^***^		0.000	1.000	0.178	−0.052
7. Turnover Intention	−0.309^***^	−0.253^***^	−0.070	−0.170^***^	−0.248^***^	−0.254^***^	0.000	1.000	−0.134	−0.216

^*^p < 0.05, ^**^ *p* < 0.01, ^***^ *p* < 0.001. SD represents standard deviation. Since all constructs in PLS-SEM are standardized, the mean is 0 and the standard deviation is 1.

All constructs were found to follow a normal distribution with skewness values within ±2 and kurtosis values within ±7 (Kline, 2005). The correlation analysis indicated significant positive correlations among most constructs. In contrast, turnover intention showed significant negative correlations with the other variables, except for hierarchy culture, for which the correlation was not statistically significant.

### Measurement model evaluation

To examine the impact of organizational culture on turnover intention among Korean Generation Z employees through the mediating effects of job satisfaction and organizational commitment, the measurement model was first evaluated. The evaluation of the measurement model depends on whether the constructs are modeled as reflective or formative. Since the research model incorporates both reflective and formative constructs, evaluations for both types were conducted.

For the reflective model, the assessment was performed using reliability and validity measures. Reliability was evaluated in terms of indicator reliability and internal consistency, while validity was examined through convergent and discriminant validity. The reflective constructs in this study included adhocracy culture, clan culture, hierarchy culture, market culture, organizational commitment, and turnover intention. The reliability and validity assessment revealed that hierarchy culture showed a reliability issue. Specifically, two hierarchy culture items—“Our company has a top-down communication and information flow” and “Our company emphasizes a sense of hierarchy between supervisors and subordinates” had low outer loadings of 0.443 and 0.393, respectively. In exploratory research, external loadings of.400 or higher are considered acceptable (Hulland, 1999), while it is common practice to remove items with external loadings below this threshold (Hair et al., 2011). In fact, previous studies that utilized the hierarchy culture items from HCCP II have either deleted some of the hierarchy culture items (Lee, 2017) or used only a representative item (Cho, 2013). However, this study retained these items, considering that they effectively capture the unique characteristics of hierarchy culture as conceptualized by Kimberly and Quinn (1984), so removing them could weaken content validity. Instead of deleting these problematic items, hierarchy culture was restructured into two sub-dimensions—norm-oriented culture and rank-oriented culture— to address potential measurement limitations while preserving content validity, following the approach suggested by Hair et al. (2022). Furthermore, the item “Our company emphasizes formal procedures, rules and policies,” which had a high outer loading of 0.971, could be identified as representative of norm-oriented culture, while the two lower-loading items could be categorized under rank-oriented culture. Accordingly, a higher-order construct specification was applied to hierarchy culture.

The reliability results for the first-order reflective model, considering the above circumstances, are presented in Table 4. Examining indicator reliability based on all constructs except for the single construct (i.e., turnover intention), all indicators generally showed outer loadings of 0.7 or higher, thereby meeting the criterion for indicator reliability. Although some items in market culture and organizational commitment had outer loadings below 0.7, they remained above 0.4, which is considered acceptable as long as internal consistency and convergent validity are satisfied (Hair et al., 2022). In terms of internal consistency, Cronbach's alpha values for rank-oriented culture, market culture, and organizational commitment ranged between 0.6 and 0.7, falling within the acceptable range. Moreover, composite reliability (CR) values exceeded 0.7, confirming sufficient internal consistency across all constructs. Overall, the measurement model demonstrated generally acceptable reliability and validity, supporting the suitability of the research model for further structural analysis.

**Table 4:** Constructs reliability and convergent validity of the reflective measurement model (First-order model).

Constructs	Indicator	Outer Loading	Cronbach's α	CR	AVE
Adhocracy culture	Adhocracy culture1	0.848	0.765	0.864	0.679
	Adhocracy culture2	0.857			
	Adhocracy culture3	0.765			
Clan culture	Clan culture1	0.849	0.759	0.861	0.674
	Clan culture2	0.786			
	Clan culture3	0.827			
Norm-oriented culture	Hierarchy culture1	1.000	1.000	1.000	1.000
Rank-oriented culture	Hierarchy culture2	0.915	0.614	0.831	0.713
	Hierarchy culture3	0.767			
Market culture	Market culture1	0.535	0.627	0.786	0.559
	Market culture2	0.813			
	Market culture3	0.855			
Organization commitment	Organization commitment1	0.670	0.688	0.821	0.608
	Organization commitment2	0.779			
	Organization commitment3	0.877			
Turnover intention	Turnover intention	1.000	1.000	1.000	1.000

CR and AVE represent Composite Reliability and Average Variance Extracted, respectively. Since reliability and validity cannot be assessed for a single construct, all values are recorded as 1.

Upon examining the validity of the first-order reflective model, as shown in Table 4, all constructs demonstrated AVE values above 0.5, confirming convergent validity. Regarding discriminant validity, the Fornell-Larcker criterion results in Table 5 indicated that the square root of each construct's AVE was greater than its correlations with other constructs, supporting the establishment of discriminant validity. According to Table 6, all HTMT values were below the recommended threshold of 0.85. Although the HTMT value between adhocracy culture and clan culture approached the threshold, it remained below the recommended cutoff, suggesting acceptable discriminant validity.

**Table 5:** Fornell-Larcker criterion results for the reflective measurement model (first-order model).

Constructs	1	2	3	4	5	6	7
1. Adhocracy Culture	**0.824**						
2. Clan Culture	0.624	**0.821**					
3. Norm-oriented Culture	0.419	0.506	1.000				
4. Rank-oriented Culture	0.104	0.108	0.270	**0.844**			
5. Market Culture	0.536	0.523	0.460	0.275	**0.748**		
6. Organization Commitment	0.481	0.484	0.228	0.047	0.379	**0.780**	
7. Turnover Intention	−0.309	−0.253	−0.086	0.060	−0.170	−0.253	1.000

The square root of the AVE value for each construct is bolded. Since validity cannot be assessed for a single construct, its square root AVE value is recorded as 1.

**Table 6:** HTMT results for the reflective measurement model (first-order model).

Constructs	1	2	3	4	5	6	7
1. Adhocracy culture	-						
2. Clan culture	0.822	-					
3. Norm-oriented culture	**0.471**	**0.585**	-				
4. Rank-oriented culture	**0.137**	**0.226**	**0.350**	-			
5. Market culture	**0.705**	**0.705**	**0.554**	**0.559**	-		
6. Organization commitment	**0.634**	**0.635**	**0.272**	**0.101**	**0.512**	-	
7. Turnover intention	**0.347**	**0.287**	**0.086**	**0.079**	**0.180**	**0.276**	-

HTMT values for which the 95% bias-corrected bootstrap confidence interval does not include 0.85 are bolded. HTMT values for which the 95% bias-corrected bootstrap confidence interval does not include 0.90 are shaded.

Next, the reliability results for the second-order reflective model based on hierarchy culture are presented in Table 7. The indicator reliability for rank-oriented culture was initially low (outer loading of 0.452), but indicator reliability became more acceptable under the higher-order construct specification. Although the Cronbach's alpha did not meet the threshold, the composite reliability (CR) was 0.706, exceeding the recommended minimum of 0.7, and the AVE was 0.580, surpassing the acceptable threshold of 0.5, indicating that rank-oriented culture could be retained in the model. Discriminant validity was confirmed using the Fornell-Larcker criterion in Table 8; however, as shown in Table 9, the HTMT value between hierarchy culture and market culture was 0.919, exceeding the recommended threshold and indicating a potential lack of discriminant validity between these constructs. This high HTMT value suggests that hierarchy culture and market culture may partially overlap, particularly within the Korean organizational context where hierarchical and performance-oriented elements often coexist. Additional evidence from cross-loadings (Table 10) showed that norm-oriented and rank-oriented culture indicators loaded more strongly on their respective constructs than on others. Taken together, these results suggest that discriminant validity is only partially supported, and therefore the findings related to hierarchy and market culture should be interpreted with caution.

**Table 7:** Constructs reliability and convergent validity of the reflective measurement model (second-order model).

Second-order construct	First-order construct	Outer loading	Cronbach's α	CR	AVE
Hierarchy Culture	Hierarchy Culture_1	0.984	0.425	0.706	0.580
	Hierarchy Culture_2	0.437			

**Table 8:** Fornell-Larcker criterion results for the reflective measurement model (second-order model).

Constructs	1	2	3	4	5	6
1. Adhocracy Culture	**0.824**					
2. Clan Culture	0.624	**0.821**				
3. Hierarchy Culture_1	0.411	0.493	**0.761**			
4. Market Culture	0.536	0.523	0.481	**0.748**		
5. Organization Commitment	0.481	0.484	0.222	0.379	**0.780**	
6. Turnover Intention	−0.309	−0.253	−0.070	−0.170	−0.254	1.000

The square root of the AVE value for each construct is bolded. Since validity cannot be assessed for a single construct, its square root AVE value is recorded as 1.

**Table 9:** HTMT results for the reflective measurement model (second-order model).

Constructs	1	2	3	4	5	6
1. Adhocracy Culture	-					
2. Clan Culture	0.822	-				
3. Hierarchy Culture_1	**0.569**	0.729	-			
4. Market Culture	**0.705**	**0.705**	0.919	-		
5. Organization Commitment	**0.634**	**0.635**	**0.331**	**0.512**	-	
6. Turnover Intention	**0.347**	**0.287**	**0.141**	**0.18**	**0.276**	-

HTMT values for which the 95% bias-corrected bootstrap confidence interval does not include 0.85 are bolded. HTMT values for which the 95% bias-corrected bootstrap confidence interval does not include 0.90 are shaded.

**Table 10:** Cross loading criterion results for the reflective measurement model (second-order model).

Construct	Adhocracy culture	Clan culture	Hierarchy culture	Market culture	Organization commitment	Turnover intention
Adhocracy culture1	**0.848**	0.523	0.343	0.423	0.387	−0.227
Adhocracy culture2	**0.857**	0.504	0.413	0.451	0.406	−0.309
Adhocracy culture3	**0.765**	0.525	0.241	0.453	0.397	−0.216
Clan culture1	0.547	**0.849**	0.351	0.435	0.461	−0.259
Clan culture2	0.504	**0.785**	0.438	0.371	0.333	−0.192
Clan culture3	0.486	**0.827**	0.437	0.48	0.388	−0.165
Hierarchy culture_1	0.419	0.506	**0.984**	0.46	0.228	−0.086
Hierarchy culture_2	0.104	0.108	**0.437**	0.274	0.047	0.06
Market culture1	0.167	0.203	0.268	**0.534**	0.11	−0.014
Market culture2	0.468	0.466	0.418	**0.812**	0.301	−0.141
Market culture3	0.467	0.435	0.382	**0.855**	0.361	−0.168
Organization commitment1	0.273	0.307	0.172	0.287	**0.669**	−0.054
Organization commitment2	0.343	0.286	0.151	0.219	**0.779**	−0.224
Organization commitment3	0.467	0.497	0.198	0.369	**0.877**	−0.261
Turnover intention	−0.309	−0.253	−0.07	−0.170	−0.254	**1.000**

The highest loading of each item is bolded.

**Figure 3:**
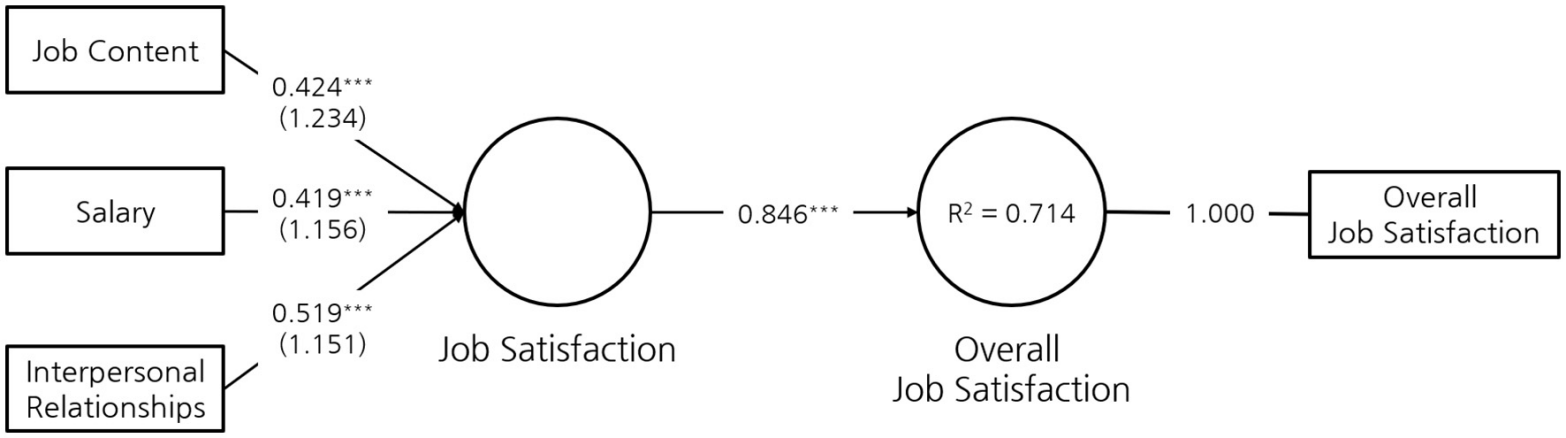
Evaluation results of the formative measurement model. ^*^p < 0.05, ^**^ *p* < 0.01, ^***^ *p* < 0.001. Circles indicate latent variables, and rectangles indicate observed (measurement) variables.

Next, the evaluation results for the formative measurement model are presented in Figure 3. In this study, job satisfaction was measured formatively. To verify whether this measurement approach was appropriate, the convergent validity of job satisfaction was examined using an overall job satisfaction item included in the same survey. The coefficient of job satisfaction on overall job satisfaction was 0.846, which was statistically significant, and the explanatory power was also high at 0.714. Additionally, all Variance Inflation Factor (VIF) values were below 3, indicating no multicollinearity issues among the job satisfaction items. Furthermore, the outer weights of all job satisfaction indicators were statistically significant. Taken together, these results provide support for the appropriateness of modeling job satisfaction as a formative construct in the present study.

### Structural model evaluation

After establishing the adequacy of the measurement model, the structural model was evaluated. Prior to testing the path coefficients, the model's explanatory power and predictive relevance were assessed.

First, as shown in Table 11, the explanatory power of the model was assessed. The effect sizes of the paths related to job satisfaction and organizational commitment ranged from 0.02, representing a small effect, to 0.15, classified as a medium effect (Cohen, 1988). In contrast, for turnover intention, most paths had effect sizes below 0.02, indicating a relatively minor impact. In summary, the relative influence of exogenous latent variables on endogenous latent variables within the model was generally small. The *R*^2^ values were 0.367 for job satisfaction, 0.365 for organizational commitment, and 0.126 for turnover intention. While the explanatory power for job satisfaction and organizational commitment can be considered moderate, the *R*^2^ value for turnover intention is relatively low. However, this is not unexpected, as turnover intention is inherently influenced by a wide range of factors at both the individual and organizational levels. In particular, prior research has shown that variables such as career opportunities and work stress play a substantial role in shaping turnover intention. In this study, the model was intentionally specified to focus on the role of organizational culture in relation to job satisfaction, organizational commitment, and turnover intention. Therefore, the relatively low *R*^2^ should be interpreted as reflecting the partial explanatory role of organizational culture, rather than indicating a deficiency in the model. These findings highlight that while organizational culture is an important factor, it represents only one part of a broader set of determinants influencing turnover intention.

**Table 11:** Explanatory power of the structural model.

Construct	Path	f^2^	R^2^
Job Satisfaction	Adhocracy culture → Job satisfaction	0.	0.367
	Clan culture → Job satisfaction	0.060	
	Hierarchy culture → Job satisfaction	0.030	
	Market culture → Job satisfaction	0.012	
Organization Commitment	Adhocracy culture → Organization commitment	0	0.365
	Clan culture → Organization commitment	0.035	
	Hierarchy culture → Organization commitment	0.023	
	Market culture → Organization commitment	0.008	
	Job satisfaction → Organization commitment	0.104	
Turnover Intention	Adhocracy culture → Turnover intention		0.126
	Clan culture → Turnover intention	0.004	
	Hierarchy culture → Turnover intention	0.011	
	Market culture → Turnover intention	0.000	
	Job satisfaction → Turnover intention	0.005	
	Organization commitment → Turnover intention	0.008	

PLS-RMSE refers to the RMSE obtained when analyzed using PLS-SEM. LM-RMSE refers to the RMSE obtained when analyzed using a linear regression model.

The results regarding predictive relevance are presented in Table 12. When comparing the predictive power of the PLS-SEM model against that of a linear regression model, it was observed that the Root Mean Squared Error (RMSE) was lower in the PLS-SEM model for all endogenous latent variables except for the interpersonal relationship item in job satisfaction. This suggests that PLS-SEM demonstrated relatively better predictive performance than the linear regression model in terms of predictive relevance (Hair et al., 2022). Additionally, the *Q*^2^ values were all greater than 0, further confirming that the structural model met the criteria for predictive relevance (Hair et al., 2022).

**Table 12:** Predictive relevance of the structural model.

Construct	Indicator	PLS-RMSE	LM-RMSE	Q^2^
Job satisfaction	Job content	0.819	0.827	0.208
	Salary	0.948	0.971	0.160
	Interpersonal relationship	0.867	0.861	0.199
Organization commitment	Organization commitment1	0.903	0.936	0.103
	Organization commitment2	0.971	0.987	0.101
	Organization commitment3	0.784	0.802	0.283
Turnover intention	Turnover intention	0.945	0.966	0.086

PLS-RMSE refers to the RMSE obtained from analysis using PLS-SEM. LM-RMSE refers to the RMSE obtained from analysis using a linear regression model.

Based on the explanatory power and predictive relevance of the structural model, the path coefficients included in the model were tested. As shown in Table 13, the organizational cultures that were significantly positively associated with job satisfaction in terms of direct effects included adhocracy culture, clan culture, and hierarchy culture. Regarding organizational commitment, adhocracy culture, clan culture, and job satisfaction showed positive direct effects, whereas hierarchy culture was negatively associated with organizational commitment. For turnover intention, only adhocracy culture demonstrated a statistically significant effect in reducing turnover intention. This suggests that adhocracy culture was the only organizational culture type that showed a significant association with turnover intention among Korean Generation Z employees.

**Table 13:** Direct effects in the structural model.

Path	Coefficient	Standard deviation	95% bootstrapped confidence interval (Bias-corrected)
AC → JS	0.202	0.063	(0.081, 0.328)
AC → OC	0.200	0.058	(0.080, 0.311)
AC → TI	−0.218	0.079	(−0.365, −0.059)
CC → JS	0.270	0.064	(0.144, 0.394)
CC → OC	0.213	0.065	(0.083, 0.339)
CC → TI	−0.083	0.084	(−0.251, 0.082)
HC → JS	0.166	0.056	(0.052, 0.273)
HC → OC	−0.149	0.055	(−0.259, −0.042)
HC → TI	0.122	0.073	(−0.024, 0.260)
MC → JS	0.112	0.061	(−0.009, 0.230)
MC → OC	0.090	0.052	(−0.014, 0.189)
MC → TI	0.012	0.067	(−0.115, 0.147)
JS → OC	0.323	0.059	(0.200, 0.433)
JS → TI	−0.111	0.070	(−0.244, 0.029)
OC → TI	−0.083	0.066	(−0.212, 0.048)

AC, CC, HC, MC, JS, OC, and TI stand for Adhocracy Culture, Clan Culture, Hierarchy Culture, Market Culture, Job Satisfaction, Organizational Commitment, and Turnover Intention, respectively. Significant paths are shaded.

The results of the mediation effect analysis, presented in Table 14, indicate that adhocracy culture, clan culture, and hierarchy culture showed significant indirect associations with organizational commitment through job satisfaction. All three indirect pathways were statistically significant and positively associated with organizational commitment through job satisfaction. However, other indirect pathways did not yield statistically significant results.

**Table 14:** Mediation effects in the structural model.

Path	Coefficient	Standard deviation	95% Bootstrapped confidence interval (Bias-corrected)
AC → JS → OC	0.065	0.024	(0.026, 0.120)
AC → JS → TI	−0.022	0.018	(−0.068, 0.002)
AC → OC → TI	−0.017	0.015	(−0.053, 0.007)
AC → JS → OC → TI	−0.005	0.005	(−0.019, 0.002)
CC → JS → OC	0.087	0.026	(0.044, 0.146)
CC → JS → TI	−0.030	0.021	(−0.077, 0.005)
CC → OC → TI	−0.018	0.016	(−0.060, 0.007)
CC → JS → OC → TI	−0.007	0.007	(−0.023, 0.003)
HC → JS → OC	0.053	0.021	(0.018, 0.103)
HC → JS → TI	−0.018	0.013	(−0.051, 0.001)
HC → OC → TI	0.012	0.012	(−0.004, 0.045)
HC → JS → OC → TI	−0.004	0.004	(−0.017, 0.001)
MC → JS → OC	0.036	0.020	(−0.001, 0.079)
MC → JS → TI	−0.012	0.011	(−0.041, 0.002)
MC → OC → TI	−0.008	0.008	(−0.032, 0.002)
MC → JS → OC → TI	−0.003	0.003	(−0.013, 0.001)
JS → OC → TI	−0.027	0.022	(−0.075, 0.014)

AC, CC, HC, MC, JS, OC, and TI stand for Adhocracy Culture, Clan Culture, Hierarchy Culture, Market Culture, Job Satisfaction, Organizational Commitment, and Turnover Intention, respectively. Significant paths are shaded.

The final analysis of the structural model focused on the total effects, as presented in Table 15. The results indicate that adhocracy culture, clan culture, and hierarchy culture were significantly and positively associated with job satisfaction. Adhocracy culture, clan culture, and market culture were significantly positively associated with organizational commitment, and an increase in job satisfaction correspondingly was significantly related to organizational commitment. Regarding turnover intention, adhocracy culture was the only organizational culture that showed a significant negative association with turnover intention. Additionally, higher job satisfaction was also associated with a lower turnover intention. These findings provide preliminary evidence that adhocracy culture and job satisfaction may be associated with lower turnover intention among the Korean Generation Z employees.

**Table 15:** Total effects in the structural model.

Path	Coefficient	Standard deviation	95% Bootstrapped confidence interval (bias-corrected)
AC → JS	0.202	0.063	(0.082, 0.329)
AC → OC	0.265	0.062	(0.143, 0.383)
AC → TI	−0.263	0.074	(−0.411, −0.119)
CC → JS	0.270	0.064	(0.147, 0.395)
CC → OC	0.300	0.067	(0.163, 0.423)
CC → TI	−0.138	0.080	(−0.293, 0.019)
HC → JS	0.166	0.056	(0.055, 0.274)
HC → OC	−0.096	0.057	(−0.211, 0.013)
HC → TI	0.112	0.072	(−0.032, 0.248)
MC → JS	0.112	0.060	(−0.009, 0.229)
MC → OC	0.126	0.055	(0.014, 0.232)
MC → TI	−0.011	0.068	(−0.142, 0.127)
JS → OC	0.323	0.058	(0.201, 0.434)
JS → TI	−0.138	0.064	(−0.262,−0.012)
OC → TI	−0.083	0.067	(−0.213, 0.049)

AC, CC, HC, MC, JS, OC, and TI stand for Adhocracy Culture, Clan Culture, Hierarchy Culture, Market Culture, Job Satisfaction, Organizational Commitment, and Turnover Intention, respectively. Significant paths are shaded.

## Summary and discussion

### Organizational culture and turnover intention among Generation Z employees

The entry of Generation Z into Korea's workforce has only recently begun, yet they are already exhibiting higher rates of job change than previous generations (Jang and Lee, 2024). This study examines the relationships among organizational culture, job satisfaction, organizational commitment, and turnover intention among Korean Generation Z employees using a structural equation modeling approach. The findings and their implications are presented below.

First, adhocracy culture showed the strongest pattern of associations with employee outcomes among Korean Generation Z employees, compared to other types of organizational culture. Specifically, adhocracy culture was significantly and positively associated with job satisfaction and organizational commitment, while showing a significant negative association with turnover intention. This finding may suggest that organizational environments characterized by flexibility and innovation may be related to more favorable employee attitudes in this context. This pattern may also reflect the possibility that innovation and flexibility are viewed favorably among younger employees in contemporary organizational environments. Consistent with this interpretation, Jang and Lee (2024) reported that many Korean MZ employees placed considerable importance on opportunities for personal growth, autonomy, and self-development when evaluating potential workplaces. These findings may provide a possible explanation for why adhocracy culture showed the strongest pattern of associations with employee attitudes in this sample.

Second, contrary to common expectations, clan culture was significantly and positively associated with job satisfaction and organizational commitment among Korean Generation Z employees. This finding suggests that interpersonal relationships and supportive work environments remain relevant in shaping employee attitudes. A sense of belonging and positive interpersonal relationships have long been recognized as important aspects of employees' workplace experiences and fundamental human needs (Lee and Kim, 2021). However, although clan culture was positively associated with job satisfaction and organizational commitment, it was not significantly associated with turnover intention. Previous studies have suggested that younger employees place considerable importance on individual well-being, career development, and personal growth (Jang and Lee, 2024; Cho et al., 2024), and Lee and Suh (2025) further found that turnover considerations among younger Korean employees were closely associated with whether expectations regarding career development opportunities, work–life balance, and organizational support were fulfilled. Therefore, supportive interpersonal relationships alone may not be sufficient to reduce turnover intention if employees perceive limited opportunities for career growth or future development. These findings suggest that the influence of clan culture on employee attitudes may be context-dependent.

Third, hierarchy culture showed a significant positive association with job satisfaction among Korean Generation Z employees, which contrasts with common expectations. In the Korean organizational context, formal procedures, clear rules, and structured systems may provide greater clarity and predictability in work processes, potentially contributing to higher levels of job satisfaction. In this regard, Deloitte (2023) reported that younger employees tend to place considerable importance on fairness and transparency in the workplace, which may help explain the positive association observed between hierarchy culture and job satisfaction. However, hierarchy culture was negatively associated with organizational commitment and showed no significant association with turnover intention, suggesting that its relationship with employee attitudes may be complex. Given the relatively low outer loadings of the hierarchy culture construct, these findings should be interpreted with caution, and future research is needed to further examine how different aspects of hierarchy culture relate to employee attitudes.

Fourth, market culture showed a limited pattern of associations, being significantly and positively related only to organizational commitment among Korean Generation Z employees. It was not significantly associated with job satisfaction or turnover intention. One possible explanation is that competitive and performance-oriented organizational environments may not necessarily be associated with higher job satisfaction or lower turnover intention among younger employees. In this regard, Jang and Lee (2024) suggested that younger employees often place considerable importance on factors such as personal well-being, work–life balance, and self-development when evaluating workplaces, which may help explain the relatively limited associations observed for market culture. Therefore, the findings related to market culture should be interpreted with caution, and further research is needed to better understand how market-oriented cultural elements relate to employee attitudes.

### Implications

From a practical and policy perspective, the findings of this study provide several preliminary implications. Organizations may consider fostering work environments characterized by autonomy, flexibility, and innovation, as adhocracy culture was positively associated with job satisfaction and organizational commitment while showing a negative association with turnover intention in this sample. In addition, supportive interpersonal relationships and a sense of belonging remained positively associated with employee attitudes, suggesting that mentoring systems and collaborative work environments may continue to be relevant in organizational settings involving younger employees. However, given the exploratory nature of the findings and the relatively low explanatory power for turnover intention, these implications should be interpreted with caution.

Additionally, this study provides several methodological implications. Unlike many previous studies, this research employed PLS-SEM and modeled job satisfaction as a formative construct. Components of job satisfaction—such as satisfaction with job content, salary, and interpersonal relationships—may represent relatively distinct aspects of the overall construct rather than interchangeable indicators. Therefore, a formative specification may provide a useful approach for examining the multidimensional nature of job satisfaction within the context of this study.

### Limitations and future research

Although the findings provide meaningful insights, this study has several limitations. First, turnover intention was measured using a single-item indicator. Single-item indicators have been used for relatively concrete and unidimensional constructs and are compatible with the use of PLS-SEM; however, this approach may still limit the ability to fully capture the complexity and variability of turnover intention. Therefore, the findings should be interpreted with caution and considered exploratory in nature. Future research is encouraged to adopt validated multi-item scales to enhance measurement reliability and robustness.

Second, some measurement instruments, particularly those related to organizational culture (e.g., hierarchy culture), showed relatively low reliability. Although this study addressed this issue by modeling hierarchy culture as a higher-order construct composed of sub-dimensions, this approach may not fully resolve limitations related to measurement precision. Therefore, the findings related to hierarchy culture should be interpreted with caution. Furthermore, future research is encouraged to refine and expand measurement items to enhance reliability, content validity, and factorial clarity.

Third, the relatively low explanatory power (R^2^) for turnover intention indicates that organizational culture explains only part of turnover intention among Korean Generation Z employees in this study. This implies that additional factors such as compensation, career development opportunities, workload or stress, and supervisory support should be incorporated in future research to provide a more comprehensive understanding. Furthermore, this study focused exclusively on Generation Z employees to examine these relationships within a Generation Z sample. However, given the absence of cross-generational comparisons, caution is needed in interpreting the findings as specific to Generation Z. Future research is encouraged to conduct cross-generational comparisons to examine whether the observed relationships between organizational culture and turnover intention are generation-specific or generalizable across different cohorts.

Finally, the sample in this study was drawn exclusively from organizations with 100 or more employees, as the dataset was originally designed to include only organizations with 100 or more employees. Therefore, the findings may not be generalizable to smaller firms. Given the absence of data on organizations with fewer than 100 employees, it was not possible to examine differences across firm sizes in the present study. Future research is encouraged to incorporate data from smaller firms to examine whether the observed relationships differ across organizations of varying sizes and industry sectors.

### Conclusion

As Generation Z continues to enter the labor market, understanding the workplace experiences of younger employees has become increasingly important in the Korean context. This study examined associations among organizational culture, job satisfaction, organizational commitment, and turnover intention among Korean Generation Z employees. Among the four culture types, adhocracy culture showed the most consistent pattern of favorable associations with employee attitudes, whereas the relationships observed for clan, hierarchy, and market cultures were more limited or context-dependent. The findings provide exploratory evidence regarding how organizational culture is related to employee attitudes in this sample. Overall, this study offers preliminary insights that may serve as a basis for future research examining employee attitudes and organizational experiences among younger workforce groups.

## Data Availability

Publicly available datasets were analyzed in this study. This data can be found here: The data analyzed in this study are from the Human Capital Corporate Panel (HCCP II), provided by the Korea Research Institute for Vocational Education and Training (KRIVET). The dataset is not publicly downloadable but can be accessed upon reasonable request through the official KRIVET website (https://www.krivet.re.kr/eng/).
